# Utility of real-time prospective motion correction (PROMO) on 3D T1-weighted imaging in automated brain structure measurements

**DOI:** 10.1038/srep38366

**Published:** 2016-12-05

**Authors:** Keita Watanabe, Shingo Kakeda, Natsuki Igata, Rieko Watanabe, Hidekuni Narimatsu, Atsushi Nozaki, Dan Rettmann, Osamu Abe, Yukunori Korogi

**Affiliations:** 1 Department of Radiology, University of Occupational and Environmental Health School of Medicine, Japan; 2 MR Applications and Workflow Asia Pacific, GE Healthcare, Japan; 3 MR Applications and Workflow, GE Healthcare, Rochester, MN, USA; 4 Department of Radiology, Nihon University School of Medicine, Japan

## Abstract

PROspective MOtion correction (PROMO) can prevent motion artefacts. The aim of this study was to determine whether brain structure measurements of motion-corrected images with PROMO were reliable and equivalent to conventional images without motion artefacts. The following T1-weighted images were obtained in healthy subjects: (A) resting scans with and without PROMO and (B) two types of motion scans (“side-to-side” and “nodding” motions) with and without PROMO. The total gray matter volumes and cortical thicknesses were significantly decreased in motion scans without PROMO as compared to the resting scans without PROMO (*p* < 0.05). Conversely, Bland–Altman analysis indicated no bias between motion scans with PROMO, which have good image quality, and resting scans without PROMO. In addition, there was no bias between resting scans with and without PROMO. The use of PROMO facilitated more reliable brain structure measurements in subjects moving during data acquisition.

Automated brain structure analysis using three-dimensional T1-weighted imaging (3D-T1WI) with voxel-based morphometry (VBM) and surface-based morphometry has contributed to the identification of morphological abnormalities in dementia and various psychiatric disorders^1^,^2^. Further, automated brain structure measurements have been applied in clinical practice to supplement assessment of brain atrophy and diagnosis of degenerative diseases^3^,^4^,^5^,^6^. However, the comparatively long acquisition time of 3D-T1WI frequently leads to the formation of motion artefacts, which can lead to mischaracterisation of the size and tissue properties of brain structures^7^,^8^. Further, Reuter *et al*. revealed that motion artefacts apparently reduced gray matter (GM) volume and cortical thickness^9^, which can lead to false results in automated brain structure measurements. Therefore, a method to reduce the incidence of motion artefacts is strongly desired.

An image-based framework for prospective motion correction, called “PROMO” (PROspective MOtion correction), was recently introduced to reduce the incidence of motion artefacts in 3D-T1WI. PROMO utilises three orthogonal 2D spiral navigator (S-NAV) acquisitions along with a flexible image-based tracking method for real-time motion measurement and correction^10^. These navigators, which are interspersed within the T1 recovery time, are used to fix the coordinate system relative to the subject brain position to correct for both in-plane and through-plane movement. Furthermore, PROMO uses non-iterative recursive Kalman filters (EKF), which are well suited for rapid real-time implementation. These features facilitate the combination of computation and correction to retrieve real-time position information with motion trajectory data to measure the position of the subject in the upcoming acquisition and to automatically rescan images acquired during intervals with significant head motion^8^,^10^.

Previous studies reported that PROMO reduced artefacts in 3D-T1WI caused by head motions of school-age children^8^,^11^. Likewise, the results of the present study revealed that PROMO allows for improved image quality in “uncooperative” populations. Since brain structure measurements are affected by a variety factors, such as pulse sequence^12^, scanner upgrade^13^ and strength field^12^,^13^,^14^, it is important to use motion-corrected images in place of conventional images to investigate the reliability of PROMO without motion artefacts for brain structure measurements. Further, because small motions at rest during acquisition may affect brain structure measurements^9^, the hypothesis of this study was that PROMO will improve the repeatability of brain structure measurements at rest. In this study, resting scans, while the subjects were explicitly trying to hold still, and prescribed motion scans with and without PROMO were acquired. The primary aim of this study was to determine whether motion-corrected images provide reliable brain structure measurements equivalent to conventional images without motion artefacts. The second aim was to assess whether PROMO improves the repeatability of brain structure measurements by correcting for small motions at rest.

## Patients and Methods

### Approval

Human experiments were carried out in accordance with the guidelines provided and approved by the Institutional Review Board of the University of Occupational and Environmental Health School of Medicine (Kitakyushu, Fukuoka, Japan).

### Subjects

The study cohort included seven healthy subjects (#1–7), consisting of four men and three women, aged 30–35 years. All participants provided written informed consent for participation in this study.

### Data acquisition

Details of scan acquisitions are summarised in Table 1. First, the following 3D-T1WI scans were obtained of subject #1 in one scanning session: (1) resting scans with and without PROMO, where the subject was asked to remain as still as possible, and (2) two types of motion scans (termed “side-to-side” and “nodding”) with and without PROMO, where the subject was asked to repeat specific predefined motions. The motions were defined as a 10-degree “side to side” rotation (right side, 5 degrees; left side, 5 degrees) and a 10-degree “nodding” rotation. One rotation was performed quickly at 20-s intervals. The subject was trained to repeat the same motions using extended EKF motion estimates. During each scan, the head of the subject rested on a pillow, stabilised by foam blocks on both sides. The subject was positioned in the bore, such that the junction of the top of the nose and the brow was at the isocentre. Scans were acquired 10 times within 3 weeks. The two types of motion scans without PROMO were acquired only five times each. The subject was blinded to whether a given scan was being acquired with or without PROMO.

Second, five resting scans with and without PROMO of two subjects (#2 and #3) were acquired on the same day.

Third, in five subjects (#3–7), one scan in each of the resting scans without PROMO and two types of motion scans with PROMO were acquired on the same day. After acquisitions, a neuroradiologist immediately checked image quality. To obtain good quality motion-corrected images, each motion scan was repeated until the image quality of the scan was judged as adequate (see “Qualitative assessments”). Quantitative motion measurements during acquisition were also confirmed.

### Magnetic resonance imaging protocol

Magnetic resonance imaging (MRI) data were obtained using a 3.0-Tesla scanner (Discovery MR750w 3.0 T Wide Bore MRI; GE Healthcare, Milwaukee, WI, USA) with a Magnetization-Prepared Rapid Acquisition Gradient Echo (MPRAGE) pulse sequence provided by GE Healthcare. The following parameters were used in scans with and without PROMO: repetition time, 2,300 ms; inversion time, 900 ms; echo time, 3 ms; flip angle, 8°; bandwidth, 31 kHz; field of view, 256 mm; section thickness, 1.0 mm; matrix, 256 × 256; and imaging time; 5 min 24 sec. If significant head motion was detected during scans with PROMO, the acquisition time was increased to rescan motion-corrupted data. Rescans were set to be triggered by a norm of ≥1 mm/degree in motion during acquisition^10^.

All images were corrected for image distortion using Gradwarp, which is a system for specific correction of image geometry distortion due to gradient non-linearity^13^.

### Qualitative assessments

Two neuroradiologists, who were blinded to PROMO status (with vs. without) and motion status (resting vs. motion), rated image quality using a three-point scale as follows: adequate = little or no detectable motion artefact with adequate image quality for brain morphometry analysis; inadequate = detectable motion artefacts with inadequate image quality for brain morphometry analysis; and poor = extreme motion artefacts.

### Quantitative assessments

#### FreeSurfer

Freesurfer v5.3.0^15^ was used for image processing. For all scans, “recon-all” and Longitudinal Processings^16^,^17^ were performed first. For subject #1, templates for one scanning session were generated from the following paired scans: a resting scan without PROMO and (A) a resting scan with PROMO; (B, C) “side to side” and “nodding” motion scans with PROMO rated as adequate; (D) a combination of “side to side” and “nodding” motion scans with PROMO rated as inadequate; and (E, F) “side to side” and “nodding” motion scans without PROMO, respectively. For subjects #2 and 3, templates were generated from each paired scan, i.e., resting scan with and without PROMO (#1), …, with and without PROMO (#5), respectively. For subjects #3–7, templates for the “side to side” and “nodding” motion scans with PROMO were constructed in the same way as for subject #1. The motivation behind the use of Longitudinal Processing was to generate topologically equivalent surface meshes for any volume under comparison. The surface mesh generated from the unbiased within-subject template was used for re-positioning of the surface mesh relative to each volume. The re-positioning procedure provided surfaces with the same geometry. Therefore, Longitudinal Processing may provide a more accurate estimate of differences^16^,^17^,^18^. Although the results provided by Longitudinal Processing may not be generalised to those provided with a conventional stream, the same differences could be detected by pooling measurements across a large population to average the processing bias^18^. After Longitudinal Processing, total GM volumes were calculated. Further, to assess differences in cortical thickness at each location in the cortex, the difference in cortical thickness was calculated for all paired scans. Then, these cortical differences were mapped on a common surface space and the average difference in cortical thickness was calculated. This process was described in detail by Fujimoto *et al*.^18^.

Second, repeatability of cortical structure measurements in the resting scans with and without PROMO of subjects #1–3 was analysed. A template was generated from the resting scans with and without PROMO, respectively. Longitudinal Processing was performed with the resting scans with and without PROMO, respectively. For subject #1, the difference in cortical thickness was calculated between two scans acquired in two continuous sessions (i.e., resting scan in session #1 vs. session #2, …, session #9 vs. session #10). For subjects #2 and #3, the difference in cortical thickness was calculated between each two continuous scans (i.e., scan #1 vs. scan #2, …, scan #4 vs. scan #5, scan #5 vs. scan #1). The absolute means and standard deviations of cortical thickness differences were calculated.

For reliability and repeatability analyses, minimal manual editing was performed, including correction of errors in the cortical reconstructions.

#### Statistical parametric mapping

The “segment” algorithm in Statistical Parametric Mapping (SPM) 12 was used^19^,^20^. The images in native space were spatially normalised, segmented into GM, white matter and cerebrospinal fluid images, and modulated for intensity using the Diffeomorphic Anatomical Registration Through Exponential Lie Algebra (DARTEL)^20^. To preserve GM volumes within each voxel, we modulated the images using the Jacobean determinants derived from the spatial normalization by DARTEL. Modulated images without smoothing were used to calculate total GM volumes based on the International Consortium for Brain Mapping template with WFU PickAtlas, version 3.0.5^21^,^22^.

#### Functional MRI of the Brain Software Library

Functional MRI of the Brain (FMRIB) Software Library (FSL) v5.0.4 (http://fsl.fmrib.ox.ac.uk/fsl/fslwiki/first) was used as the brain extraction tool (with –f 0.2 and –B option) and FMRIB’s Automated Segmentation Tool (FAST) was used for GM segmentation^23^,^24^. Total GM volumes were then calculated by multiplying the number of voxels by the voxel volume.

### Statistical analysis

The inter-observer variability in subjective ratings by two neuroradiologists was calculated as a κ value. The strength of agreement was considered fair for κ values of 0.21–0.40, moderate for κ values of 0.41–0.60, good for κ values of 0.61–0.80 and excellent for κ values of ≥0.81. After independent interpretations, any differences in assessments were resolved by consensus. The chi-squared test was used to assess the statistical significance of the different ratings.

For the quantitative assessment in total GM volumes, statistically significant differences were examined using the paired Student’s *t*-test. Resting scans without PROMO and (A) resting scans with PROMO were compared; (B, C) the “side to side” and “nodding” motion scans with PROMO were rated as adequate; (D) the combination of “side to side” and “nodding” motion scans with PROMO were rated as inadequate; and (E, F) the “side to side” and “nodding” motion scans without PROMO, respectively. Further, the distributions of the volume differences plotted against the volume means were examined by Bland–Altman analysis^25^. For repeatability analyses, the Mann–Whitney *U* test was used to compare the equality of variances in the absolute means of thickness differences.

All statistical analyses were performed with R statistical software (v3.1.0; R Foundation for Statistical Computing, Vienna, Austria). A probability (*p*) value of <0.05 was considered statistically significant.

## Results

### Imaging time of scans with PROMO

The additional acquisition times (mean ± SD) required for rescanning motion-corrupted data in the “side to side” and “nodding” motion scans with PROMO were 57.0 ± 6.1 and 59.1 ± 6.4 s for subject #1 and 58.6 ± 9.1 and 54.1 ± 10.3 s for subjects #3–7, respectively. No rescan was triggered for any resting scan with PROMO.

### EKF motion estimates for the scans with PROMO

**Figure 1:**
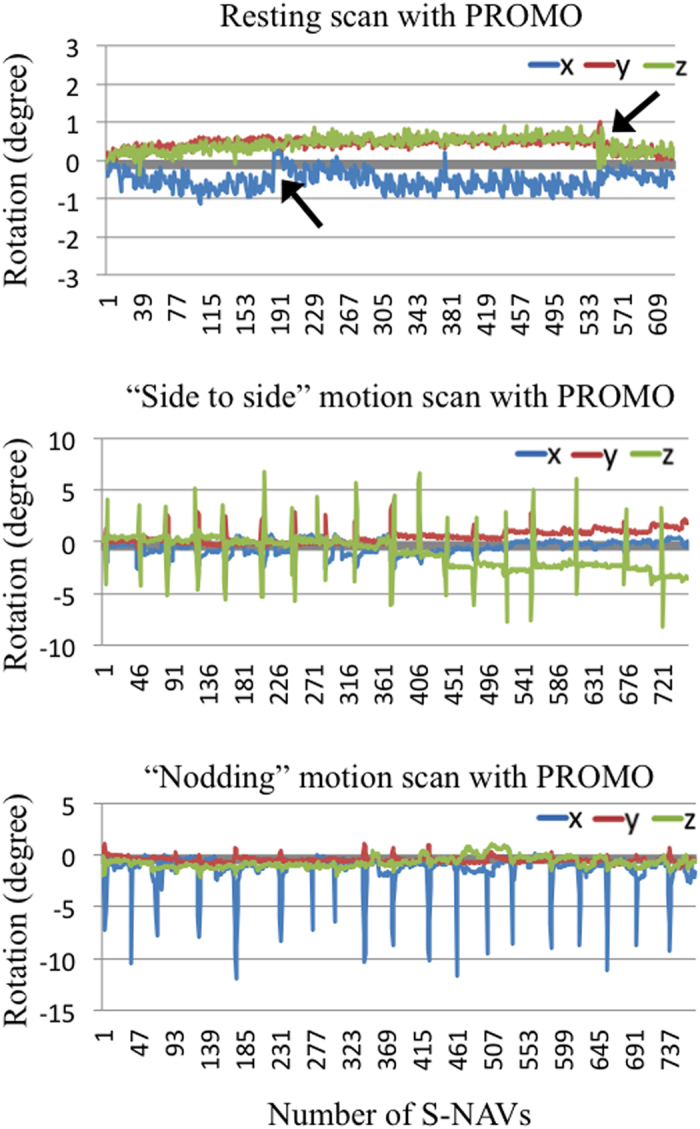
Kalman filters motion estimates for scans with PROMO. Line graphs show head rotation during scans with PROMO. Blue, red and yellow-green lines correspond, respectively, to rotation around the x-axis (left-right direction), y-axis (anterior-posterior direction) and z-axis (inferior-superior direction). The motions in Fig. 1 were measured during scans shown in Fig. 2. For the “side to side” and “nodding” motion scans, similar motions were repeated periodically. For the resting scans with PROMO, regular small motions and sudden motions (rotations of less than one degree) (arrows) were observed, even though the subject was asked to remain as still as possible.

Representative quantitative motion measurements obtained during the scans with PROMO are shown in Fig. 1. For the “side to side” and “nodding” motion scans with PROMO, about 10-degree “side to side” and “nodding” rotations were repeated periodically. For the resting scans with PROMO, although the subject was asked to remain as still as possible, small motions (rotations of less than one degree) were observed.

### Qualitative assessments

**Figure 2:**
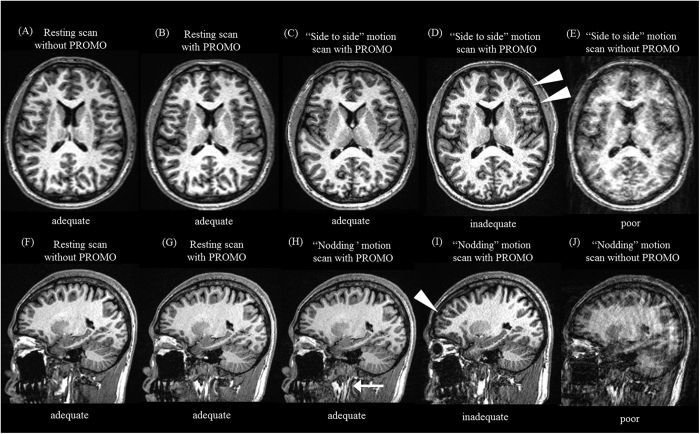
Images of resting and motion scans with and without PROMO. (**A**,**F**) Axial and sagittal images of a resting scan without PROMO. (**B,G**) Axial and sagittal images of a resting scan with PROMO. (**C,D**) Axial images of “side to side” motion scans with PROMO rated as adequate and inadequate. (**E**) An axial image of a “side to side” motion scan without PROMO. (**H**,**I**) Sagittal images of “nodding” motion scans with PROMO rated as adequate and inadequate. (**G**) A sagittal image of a “nodding” motion scan without PROMO. The overall image quality of the motion scans with PROMO rated as adequate (**C,H**) was equivalent to that of the resting scan without PROMO (**A,F**). Although motion artefacts can be seen in the “side to side” and “nodding” motion scans with PROMO rated as inadequate (**D,I**) (arrowhead), motion artefacts were reduced, as compared to the motion scans without PROMO (**E,J**). A motion artefact in the neck region (arrow) can be seen on a “nodding” motion scan with PROMO rated as adequate (**H**) because the imaging volume updates used by PROMO only accounted for rigid-body motion and the neck was not moving as a rigid body.

The image qualities are shown in Table 1. All of the resting scans without PROMO (Fig. 2A,F) and with PROMO (Fig. 2B,G) were rated as adequate in all subjects. For subject #1, all “side to side” and “nodding” motion scans without PROMO were rated as poor (Fig. 2E,J). Conversely, of the 10 “side to side” and 10 “nodding” motion scans with PROMO, eight and eight were rated as adequate, respectively (Fig. 2C,H). The remaining two “side to side” and two “nodding” motion scans with PROMO were rated as inadequate (Fig. 2D,I). The rate of adequate “side to side” and “nodding” motion scans were significantly higher with PROMO than without (*p* < 0.05).

The κ value for inter-observer variability between two radiologists was 0.98, which corresponded to excellent inter-observer agreement.

For subjects #3–7, two “side to side” and two “nodding” motion scans with PROMO were rated as inadequate. Therefore, these scans were reacquired. All of the reacquired motion scans with PROMO were rated as adequate.

### Reliability analysis of total GM volume measurements

The results of reliability analysis of total GM volume measurements are shown in Table 2.

#### (A) Resting scans with PROMO

There was no significant difference in total GM volumes in resting scans with and without PROMO for subjects #1–3 (FreeSurfer: subject #1, *p* = 0.71; subject #2, *p* = 0.71; subject #3, *p* = 0.57, SPM12: subject #1, *p* = 0.45; subject #2, *p* = 0.78; subject #3, *p* = 0.61, FSL: subject #1, *p* = 0.78; subject #2, *p* = 0.94; subject #3, *p* = 0.98).

#### (B, C) “Side to side” and “nodding” motion scans with PROMO rated as adequate

There was no significant difference in total GM volumes in “side to side” and “nodding” motion scans with PROMO rated as adequate, as compared to resting scans without PROMO for subjects #1 and #3–7 (“side to side” motion scans with PROMO rated as adequate, FreeSurfer: subject #1, *p* = 0.29; subjects #3–7, *p* = 0.76, SPM12: subject #1, *p* = 0.73; subjects #3–7, *p* = 0.34, FSL: subject #1, *p* = 0.36; subjects #3–7, *p* = 0.32, “nodding” motion scans with PROMO rated as adequate, FreeSurfer: subject #1, *p* = 0.22; subjects #3–7, *p* = 0.89, SPM12: subject #1, *p* = 0.56; subjects #3–7, *p* = 0.40, FSL: subject #1, *p* = 0.30; subjects #3–7, *p* = 0.38).

#### (D) The combination of “side to side” and “nodding” motion scans with PROMO rated as inadequate

The total GM volume in motion scans with PROMO rated as inadequate was significantly underestimated, as compared to resting scans without PROMO for subject #1 and #3–7 (FreeSurfer: subject #1, *p* = 0.04; subjects #3–7, *p* = 0.02, SPM12: subject #1, *p* < 0.01; subjects #3–7, *p* = 0.01, FSL: subject #1, *p* = 0.04; subjects #3–7, *p* = 0.03).

#### (E, F). “Side to side” and “nodding” motion scans without PROMO

Total GM volume was significantly underestimated in motion scans without PROMO, as compared to resting scans without PROMO for subject #1 (“side to side” motion scans without PROMO, FreeSurfer: subject #1, *p* < 0.01, SPM12: subject #1, *p* < 0.01, FSL: subject #1, *p* = 0.04, “noding” motion scans without PROMO, FreeSurfer: subject #1, *p* < 0.01, SPM12: subject #1, *p* < 0.01, FSL: subject #1, *p* < 0.01).

**Figure 3:**
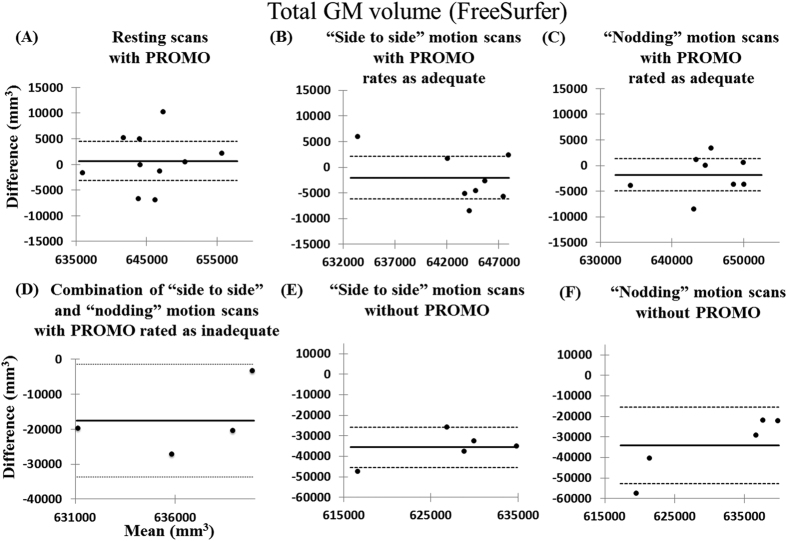
Bland–Altman analyses of the agreement with the resting scans without PROMO. In the Bland–Altman analysis of subject #1, the graphs of the total GM volumes measured by FreeSurfer for (**A**) the resting scans with PROMO, (**B**) the “side to side” motion scans with PROMO rated as adequate, (**C**) the “nodding” motion scans with PROMO rated as adequate, (**D**) the combination of the “side to side” and “nodding” motion scans with PROMO rated as inadequate, (**E**) the “side to side” motion scans without PROMO and (**F**) the “nodding” motion scans without PROMO were plotted against their differences and were compared to the resting scans without PROMO. Results of SPM12 and FSL are shown in Supplemental Fig. S1. Solid lines correspond to the mean difference. Dashed lines correspond to the mean difference ± 1.96 standard deviations and the 95% confidence interval.

Bland–Altman plots of total GM volumes for subject #1 are shown in Fig. 3 and Supplemental Fig. S1. Bland–Altman plots show the distribution of volume differences relative to volume means for six types of scans and the day-matched resting scans without PROMO. For (A) resting scans with PROMO and (B, C) “side to side” and “nodding” motion scans with PROMO rated as adequate, the volume differences were nearly symmetrically distributed around zero. Furthermore, the signed difference means were not significantly different from zero, indicating that there was no bias in the resting scans without PROMO in (A) the resting scans with PROMO and (B, C) the “side to side” and “nodding” motion scans with PROMO rated as adequate. Conversely, total GM volumes were significantly underestimated in (D) the combination of “side to side” and “nodding” motion scans with PROMO rated as inadequate and (E, F) the “side to side” and “nodding” motion scans without PROMO, as compared to the resting scans without PROMO. For subjects #2 and #3, the volume differences were nearly symmetrically distributed around zero in the comparison between the resting scans with and without PROMO (Supplemental Fig. S2). For the “side to side” and “nodding” motion scans with PROMO rated as adequate and the combination of “side to side” and “nodding” motion scans with PROMO rated as inadequate, the results for subjects #3–7 were similar to those of subject #1 (Supplemental Fig. S3).

### Reliability analysis with cortical thickness difference maps

**Figure 4:**
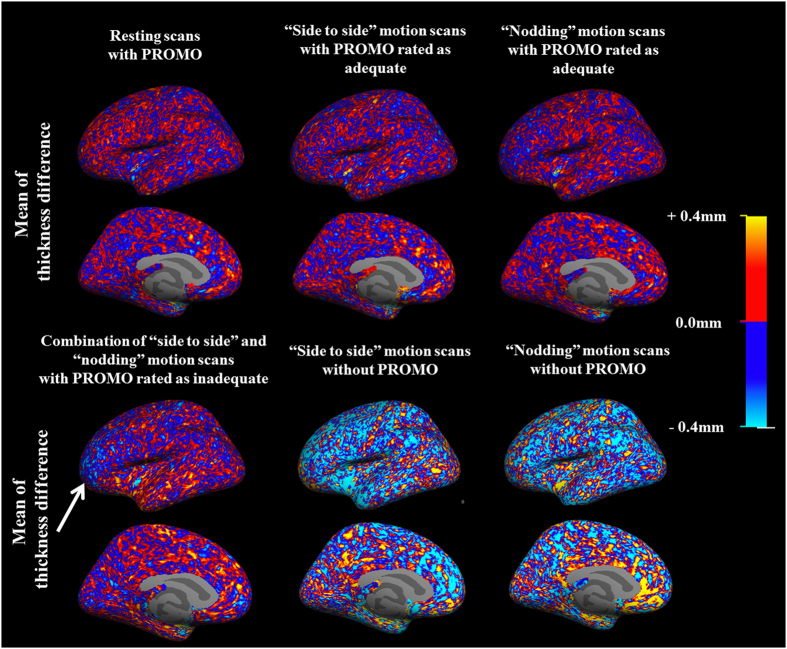
Cortical thickness difference maps. The cortical thickness difference maps of the left hemisphere in subject #1 are shown. The color maps indicate the mean thickness difference between each scan and the resting scan without PROMO acquired in the same session at each vertex on the common surface space. For the resting scans with PROMO and the “side to side” and “nodding” motion scans with PROMO rated as adequate, the spatial pattern of thickness difference is incoherent across the surface atlas, indicating no systematic discrepancies with the resting scans without PROMO. Conversely, the combination of “side to side” and “nodding” motion scans with PROMO rated as inadequate showed that the cortical thickness was underestimated, especially in the frontal cortex (arrow). The “side to side” and “nodding” motion scans without PROMO show a clear bias for thinner cortex measurements over almost the entire hemisphere. The results of the right hemisphere were similar to those of the left hemisphere.

To assess whether the patterns of thickness discrepancy with the resting scans without PROMO were consistent, the thickness differences were averaged. The results of subject #1 are presented in Fig. 4. Supplemental Figs S4 and S5 show the results of the resting scans with PROMO of subjects #2 and 3, and the motion scans with PROMO of subjects #3–7, respectively. For (A) resting scans with PROMO and (B, C) “side to side” and “nodding” motion scans with PROMO rated as adequate, the spatial pattern of thickness difference was incoherent across the surface atlas, indicating no systematic discrepancies with the resting scans without PROMO. On the other hand, for (D) the combination of the “side to side” and “nodding” motion scans with PROMO rated as inadequate, the cortical thickness in some areas was underestimated. For (E, F) the “side to side” and “nodding” motion scans without PROMO, a distinct spatial pattern was apparent. There was a clear bias for thinner cortex measurements observed over almost the entire hemisphere.

### Repeatability analysis

**Figure 5:**
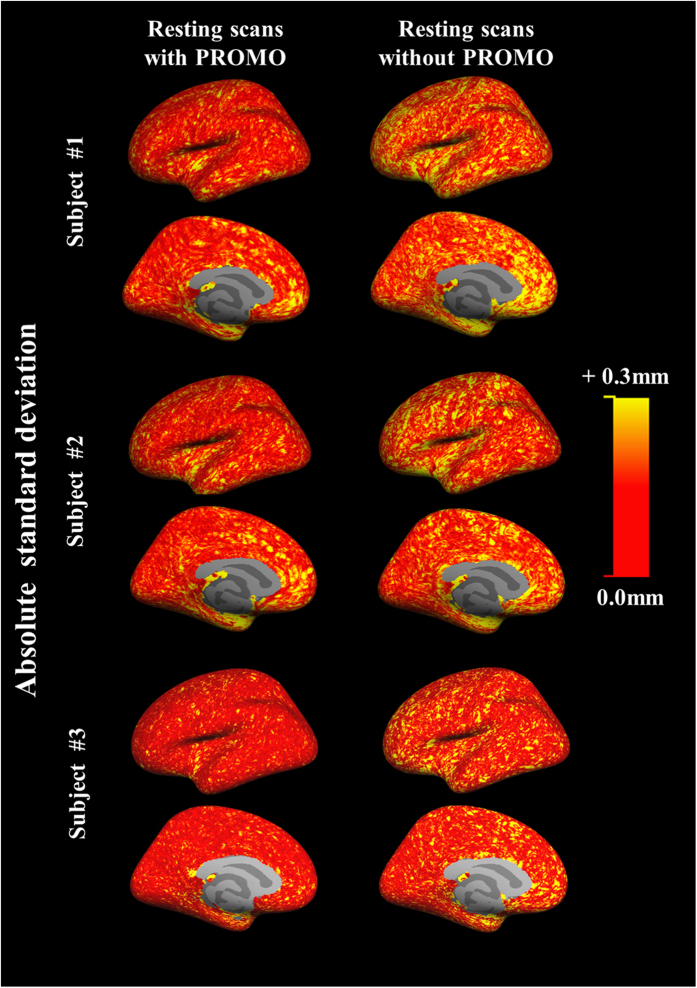
Absolute deviations of cortical thickness differences. The colour maps of the left hemisphere indicate the absolute deviations of cortical thickness differences between two continuous scans at each vertex on the common surface space. The resting scans with PROMO show smaller standard deviations over almost the entire hemisphere, as compared to the resting scans without PROMO. The results of the right hemisphere were similar to those of the left hemisphere.

The absolute mean thickness difference was less in resting scans with PROMO than without (Table 3). The absolute deviations of cortical thickness difference based on each vertex between two continuous scans are shown in Fig. 5. The absolute deviations were less in resting scans with PROMO than without over almost the entire hemisphere. These results indicated that the use of PROMO in resting scans improved repeatability of brain morphometry measurements.

## Discussion

According to the qualitative assessments conducted in this study, the use of PROMO improved image quality and reduced the incidence of motion artefacts. Further, quantitative assessments demonstrated sufficient agreement in the brain structure measurements between resting scans without PROMO and motion scans with PROMO, which were rated as adequate for brain morphometry analyses by qualitative assessments. On the other hand, total GM volume and cortical thickness were apparently underestimated in motion scans with PROMO rated as inadequate and motion scans without PROMO, which is consistent with the findings of a previous study^16^. These results suggest that, with the use of PROMO, it may be possible to decrease the number of images with unacceptable quality for brain structure measurements.

Previous studies^8^,^26^ and our study showed that motion-correction methods improve image qualities. However, not all motion artefacts were prevented by PROMO in “side to side” and “nodding” motion scans in this study. Further, the combination of “side to side” and “nodding” motion scans with PROMO rated as inadequate showed significant underestimated GM volumes compared to resting scans without PROMO in all of FreeSurfer, SPM12, and FSL analyses. These results suggest that the quality control process is important also in scans with motion-correction methods.

Scans with motion artefacts were excluded from quality control of brain structure measurements. In a previous automated brain structure measurement study, the presence of artefacts reduced the number of subjects by 15.8% among 129 Alzheimer’s disease patients, and the most common form of artefact, affecting approximately half of the rejected scans, was motion artefacts^27^. A reduction in the number of subjects has a negative effect on statistical power. Further, the exclusion of scans with motion artefacts may induce selective bias in “uncooperative” populations, such as those with dementia or Parkinson’s disease. In the case of dementia, motion artefacts may be associated with cognitive decline. Therefore, using PROMO, the increase in the number of images of acceptable quality may improve analytical precision. Although additional acquisition times for rescanning were required in the motion scans in this study, this addition is a reasonable trade-off for improved image quality.

A few previous studies have reported the utility of PROMO^8^,^11^. Of these, Brown *et al*. reported that PROMO reduced errors of the cortical surface and subcortical segmentation^8^. In that study, however, brain structure measurements were not compared between motion scans with PROMO and resting scans without PROMO because the subjects were “uncooperative” children. Therefore, the present study is the first that motion-corrected images with PROMO, which have good image qualities, could allow for reliable brain structure measurements comparable to images acquired under resting conditions. Further, it is important to note that the brain structure measurements were reliable in resting scans with PROMO. Although PROMO requires modification of the pulse sequence to include S-NAV within the T1 recovery time, the S-NAV/EKF framework did not negatively affect brain structure measurements. These results support the use of PROMO in brain structure measurements. Further, PROMO does not require additional acquisition time unless a rescan is triggered by subject movement.

Regarding motions at rest during MRI acquisitions, Reuter *et al*. reported an estimated accumulated motion of 3 mm/min in resting scans and that accumulated motion of 2 mm/min resulted in a GM volume loss of 1.4–2% in brain structure measurements^9^. In the current study, the use of PROMO in resting scans significantly improved the repeatability of cortical thickness measurements, which was likely due to motion correction for small motions at rest detected by the EKF motion estimates (Fig. 1). Small motions at rest typically result from respiration-related movements, involuntary movements and swallowing movements. PROMO prevents motion artefacts by fixing the measurement coordinate system with respect to the subject throughout the scanning process. In addition, an automated rescan triggered by a motion larger than the rescan threshold was devised to further increase the robustness of PROMO. Fixing the measurement coordinate system with respect to the subject without triggering a rescan can improve repeatability of resting scans. The results of the present study suggest that PROMO provides high-precision brain structure measurements for “cooperative” subjects as well.

Tisdall *et al*. analysed the utility of the navigator method for prospective motion correction, a research version of the vNavs on MEMPRAGE for Siemens scanner platforms, in brain morphology analyses^26^. The authors measured changes in the ratio of total brain and total GM volume of motion scans with and without motion correction compared to resting scans without motion correction using several neuroimaging methods (VBM8 of SPM8, FSL Siena and FreeSurfer) and found that the navigator method for prospective motion correction reduced motion-related bias and variance in total brain and total GM volume measurements, although comparisons of GM volume measurements on resting scans with and without PROMO were not performed. Therefore, the novelty of the present study lies in the fact that PROMO improved the repeatability of brain structure measurements, even for subjects instructed to remain as still as possible. Moreover, measurements of total GM volume and vertex-based cortical thickness, which can detect smaller differences between scans, were used in this study.

PROMO acquires data that is consistently aligned with the head. Therefore, the data acquired when the head moves relative to the distorted gradient field is not exactly consistent with the rest of the data into which it is being merged. Although an inconsistent distorted gradient field could affect local brain morphometry measurements^28^, in this study, the effect of the inconsistent distorted gradient field on the brain morphometry measurements was considered to be a minor issue from the viewpoint of good agreements between resting scans without PROMO and motion scans with PROMO. Of note, only two types of regular movements were evaluated in this study. In this instance, subjects moved back to the original position, as best he could, after the motions (Fig. 1), which might have reduced the inconsistency in the distorted gradient field. Therefore, further studies with various irregular movements are needed to investigate the effect of an inconsistent distorted gradient field on brain morphometry measurements.

There were some limitations to this study that should be addressed. First, since the data were not collected from a large cohort, it is unknown whether these results can be generalised to a larger population. Therefore, further studies with larger numbers of subjects are warranted to confirm these findings. An investigation of the relationship between motion severity and brain structure measurements in motion-corrected images is also needed. However, this relationship was not analysed in the present study because the degree of motion was not relatively significant. Second, the “side to side” and “nodding” motions in this study may differ from patient motions in clinical practice. Therefore, the use of PROMO in patients with dementia and Parkinson’s disease might be an interesting topic for further studies. In addition, comparisons of different motion correction approaches, such as the navigator method and optical tracking method, are expected. Although the optical tracking method requires implementation of a camera system, this method offers the benefit of a high frame rate and independent operation from the MRI scanning process^29^,^30^,^31^. Therefore, an optical tracking method may be more suitable than the navigator method to prevent artefacts due to fast motions, such as tremors in patients with Parkinson’s disease. Third, the absolute mean of thickness difference in the repeatability analysis (about 0.220 mm in resting scans without PROMO) was inferior to similar measurements conducted by Han *et al*. (about 0.120 mm)^14^ and Fujimoto *et al*. (about 0.140 mm)^18^. Although the reasons for these discrepancies remain unclear, differences in acquisition protocols, brain morphometry variance among subject and analytical methods should be considered.

## Conclusion

In conclusion, PROMO reduced motion-induced bias. Using PROMO, more reliable brain structure measurements were obtained in a subject who moved during data acquisition. Furthermore, PROMO improved the repeatability of brain structure measurements even in resting scans, potentially correcting for small motions at rest. In addition, because even healthy subjects are susceptible to motion artefacts, the use of PROMO should be useful in routine MRI protocols.

## Additional Information

**How to cite this article**: Watanabe, K. *et al*. Utility of real-time prospective motion correction (PROMO) on 3D T1-weighted imaging in automated brain structure measurements. *Sci. Rep.* **6**, 38366; doi: 10.1038/srep38366 (2016).

**Publisher's note:** Springer Nature remains neutral with regard to jurisdictional claims in published maps and institutional affiliations.

## Supplementary Material

Supplementary Figures

## Figures and Tables

**Table 1:** Numbers of acquisitions and image qualities.

	Resting scans without PROMO (n = 24)	Resting scans with PROMO (n = 21)	“Side to side” motion scans with PROMO (n = 18)	“Nodding” motion scans with PROMO (n = 18)	“Side to side” motion scans without PROMO (n = 5)	“Nodding” motion scans without PROMO (n = 5)	Total (n = 89)
Subject #1							
Number of acquisitions	10	10	10	10	5	5	50
Adequate/Inadequate/Poor	10/0/0	10/0/0	8/2/0	8/2/0	0/0/5	0/0/5	36/4/10
Subject #2							
Number of acquisitions	5	5	0	0	0	0	10
Adequate/Inadequate/Poor	5/0/0	5/0/0	0/0/0	0/0/0	0/0/0	0/0/0	10/0/0
Subject #3							
Number of acquisitions	6	5	1	1	0	0	13
Adequate/Inadequate/Poor	6/0/0	5/0/0	1/0/0	1/0/0	0	0	13/0/0
Subject #4							
Number of acquisitions	1	0	2	2	0	0	5
Adequate/Inadequate/Poor	1/0/0	0/0/0	1/1/0	1/1/0	0	0	3/2/0
Subject #5							
Number of acquisitions	1	0	1	1	0	0	3
Adequate/Inadequate/Poor	1/0/0	0	1/0/0	1/0/0	0	0	3/0/0
Subject #6							
Number of acquisitions	1	0	2	2	0	0	5
Adequate/Inadequate/Poor	1/0/0	0	1/1/0	1/1/0	0	0	3/2/0
Subject #7							
Number of acquisitions	1	0	1	1	0	0	3
Adequate/Inadequate/Poor	1/0/0	0/0/0	1/0/0	1/0/0	0	0	3/0/0

**Table 2:** Comparisons of total GM volumes compared to the matched resting scans without PROMO.

	Total GM volume (ml), mean ± SD			*p*		
	FreeSurfer	SPM12	FSL	FreeSurfer	SPM12	FSL
Subject #1						
Resting scans with PROMO	645.9 ± 6.2 vs. 645.3 ± 5.5	635.7 ± 0.9 vs. 635.3 ± 1.8	600.0 ± 6.0 vs. 600.5 ± 7.0	0.71	0.45	0.78
“Side to side” motion scans with PROMO rated as adequate	642.6 ± 3.7 vs. 644.7 ± 6.3	634.8 ± 1.6 vs. 636.3 ± 1.5	594.2 ± 8.6 vs. 598.7 ± 5.5	0.29	0.73	0.36
“Nodding” motion scans with PROMO rated as adequate	644.0 ± 5.8 vs. 645.8 ± 5.1	634.3 ± 1.3 vs. 634.8 ± 1.6	597.3 ± 9.1 vs. 600.0 ± 3.3	0.22	0.56	0.30
Combination of “side to side” and “nodding” motion scans with PROMO rated as inadequate	627.6 ± 7.8 vs. 645.2 ± 4.6	631.7 ± 1.0 vs. 637.2 ± 0.1	574.0 ± 9.0 vs. 604.6 ± 12.8	0.04	< 0.01	0.04
“Side to side” motion scans without PROMO	609.6 ± 5.8 vs. 645.8 ± 5.1	599.3 ± 22.9 vs. 635.9 ± 1.6	579.1 ± 13.5 vs. 596.5 ± 5.6	< 0.01	< 0.01	0.04
“Nodding” motion scans without PROMO	613.9 ± 16.9 vs. 648.0 ± 3.9	571.2 ± 26.9 vs. 635.9 ± 1.5	562.7 ± 18.0 vs. 596.5 ± 5.6	< 0.01	< 0.01	< 0.01
Subject #2						
Resting scans with PROMO	635.5 ± 3.5 vs. 635.0 ± 3.6	690.7 ± 1.9 vs. 690.3 ± 3.9	617.4 ± 4.2 vs. 617.7 ± 4.1	0.71	0.78	0.94
Subject #3						
Resting scans with PROMO	626.2 ± 2.5 vs. 624.7 ± 3.4	640.4 ± 1.4 vs. 639.7 ± 2.5	585.7 ± 4.8 vs. 585.5 ± 12.4	0.57	0.61	0.98
Subjects #3–7						
“Side to side” motion scans with PROMO rated as adequate	675.6 ± 31.1 vs. 676.3 ± 31.1	649.3 ± 42.9 vs. 651.1 ± 39.1	601.8 ± 32.3 vs. 602.5 ± 33.2	0.76	0.34	0.31
“Nodding” motion scans with PROMO rated as adequate	675.6 ± 31.9 vs. 675.8 ± 30.8	650.5 ± 37.7 vs. 651.2 ± 39.1	599.4 ± 33.2 vs. 602.5 ± 33.2	0.89	0.40	0.38
Combination of “side to side” and “nodding” motion scans with PROMO rated as inadequate	645.2 ± 15.8 vs. 651.8 ± 13.7	608.0 ± 31.2 vs. 616.2 ± 29.2	568.1 ± 25.8 vs. 578.7 ± 27.5	0.02	0.01	0.03

*The numbers next to vs. are total GM volumes of the matched resting scans without PROMO. FSL, Functional MRI of the Brain Software Library; GM, gray matter; SD, standard deviation; SPM, Statistical Parametric Mapping.

**Table 3:** Absolute mean of thickness difference in the repeatability analysis.

	Resting scan with PROMO	Resting scan without PROMO	*p*
Subject #1			
Left hemisphere	0.172 ± 0.003 mm	0.218 ± 0.025 mm	<0.01
Right hemisphere	0.166 ± 0.003 mm	0.214 ± 0.021 mm	<0.01
Subject #2			
Left hemisphere	0.193 ± 0.003 mm	0.224 ± 0.020 mm	0.03
Right hemisphere	0.193 ± 0.004 mm	0.225 ± 0.018 mm	0.03
Subject #3			
Left hemisphere	0.192 ± 0.001 mm	0.221 ± 0.018 mm	0.03
Right hemisphere	0.192 ± 0.002 mm	0.219 ± 0.017 mm	0.04
