# Supplementary material for: Utility of real-time prospective motion correction (PROMO) on 3D T1-weighted imaging in automated brain structure measurements
Source: Sci Rep. 2016 Dec 5;6:38366. doi: 10.1038/srep38366 (PMC5137153; doi:10.1038/srep38366)

Supplementary Information:

Utility of real-time prospective motion correction (PROMO) on 3D T1-weighted imaging in automated brain structure measurements

Keita Watanabe[1], Shingo Kakeda[1], Natsuki Igata[1], Rieko Watanabe[1], Hidekuni Narimatsu[1], Atsushi Nozak[2], Dan Rettmann[3], Osamu Abe[4] & Yukunori Korogi[1]

[1]Department of Radiology, University of Occupational and Environmental Health School of Medicine, Japan, [2]MR Applications and Workflow Asia Pacific, GE Healthcare, Japan, [3]MR Applications and Workflow, GE Healthcare, Rochester, MN, USA, [4]Department of Radiology, Nihon University School of Medicine, Japan

Corresponding author: Keita Watanabe

Corresponding author's address: Department of Radiology, University of Occupational and Environmental Health, 1-1 Iseigaoka, Yahatanishi-ku, Kitakyushu 807-8555, Japan.

Corresponding author's phone and fax: Tel: 093-691-7450, Fax: 093-692-0249

Corresponding author's e-mail address: sapient@med.uoeh-u.ac.jp

Supplementary Figures

Supplemental Fig. S1

Bland–Altman analyses of the agreement with the resting scans without PROMO.

In the Bland–Altman analysis of subject #1, the graphs of the total GM volumes measured by SPM12 and FSL were plotted. Solid lines correspond to the mean difference. Dashed lines correspond to the mean difference ±1.96 standard deviations and the 95% confidence interval.

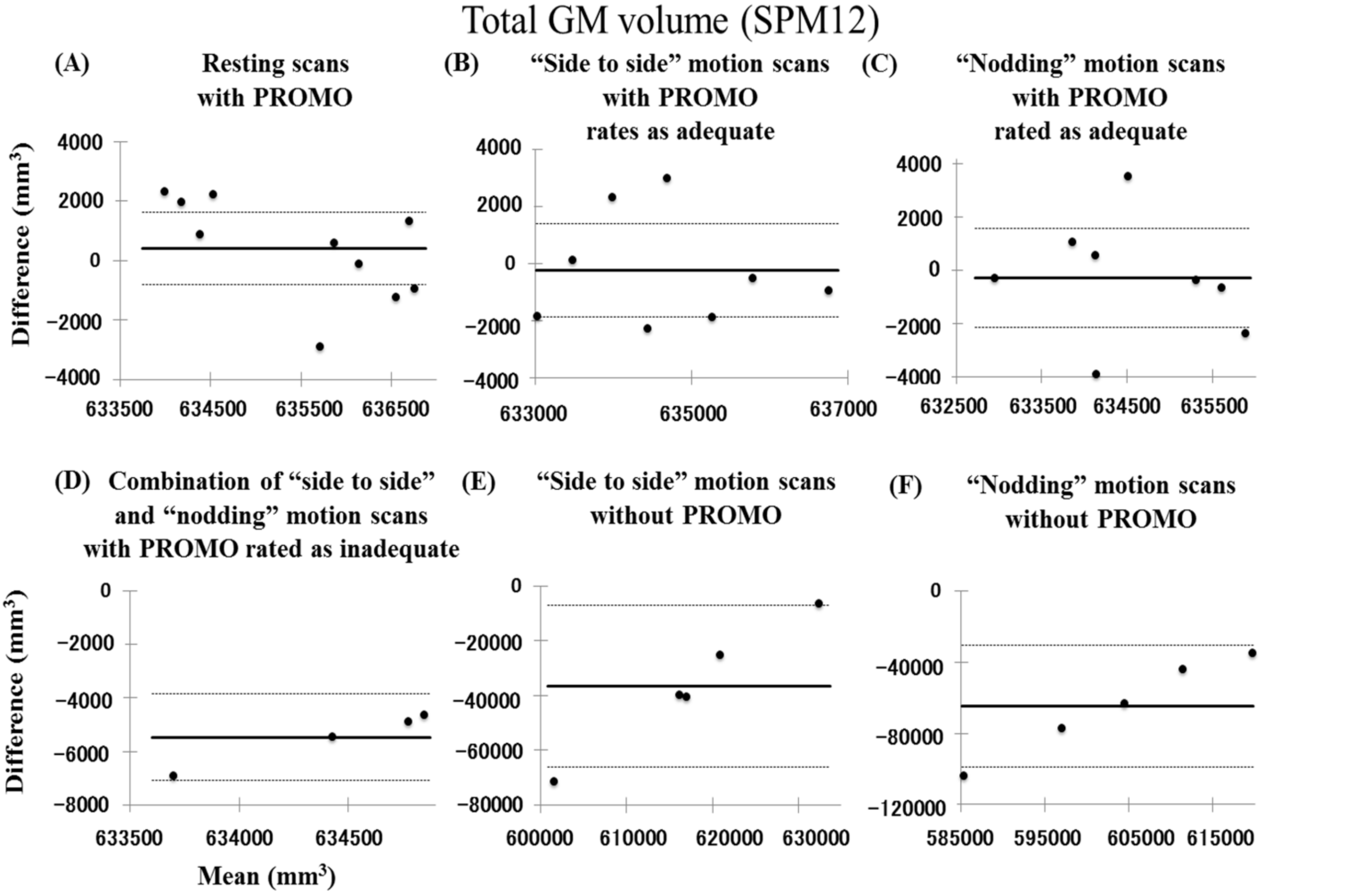

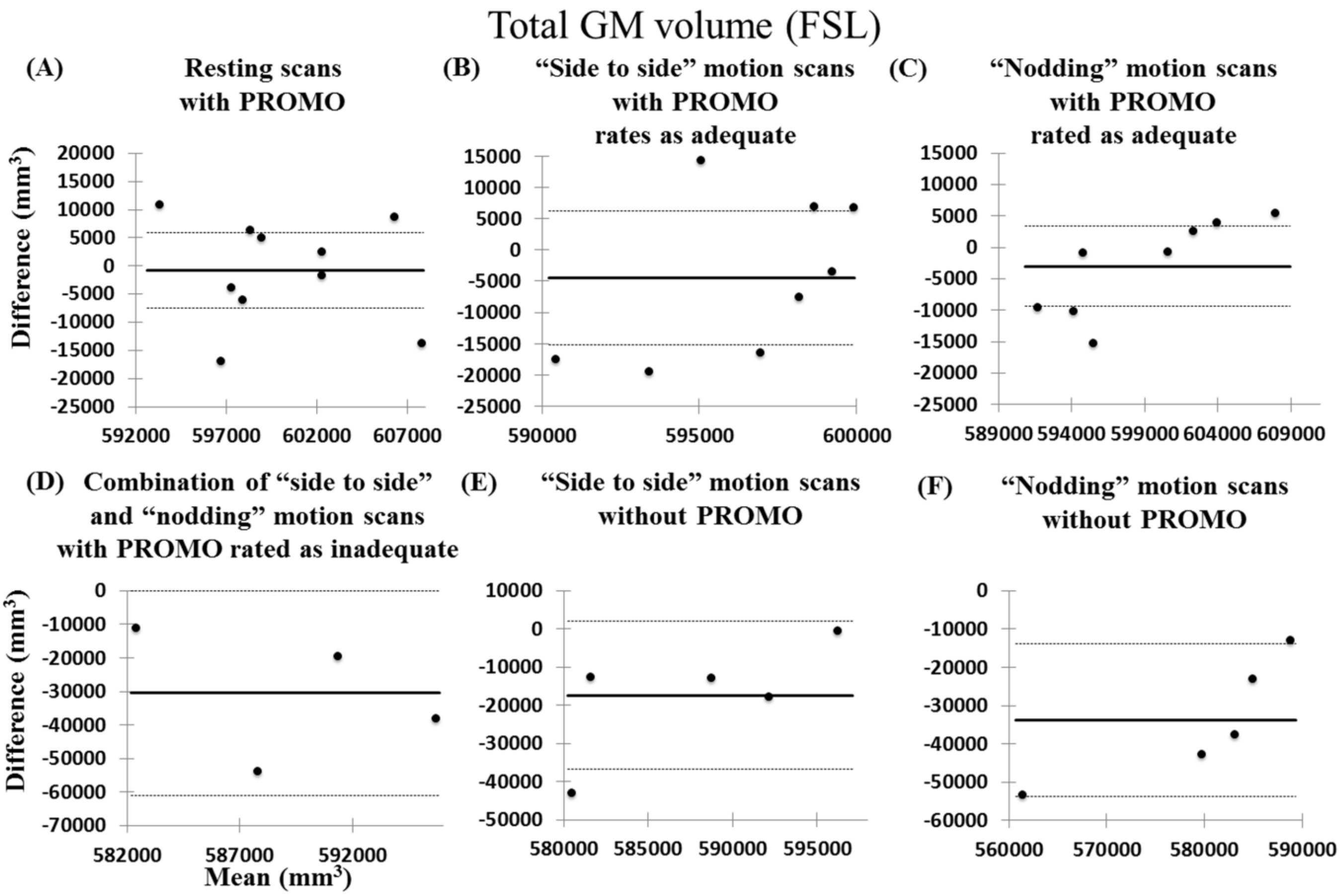

Supplemental Fig. S2

Bland-Altman analyses of the agreement between the resting scans with and without PROMO.

The Bland-Altman plots between the resting scans with and without PROMO in the subject #2 and #3 are shown. The graphs of the total GM volumes for the resting scans with PROMO were plotted against their differences and were compared to the resting scans without PROMO.

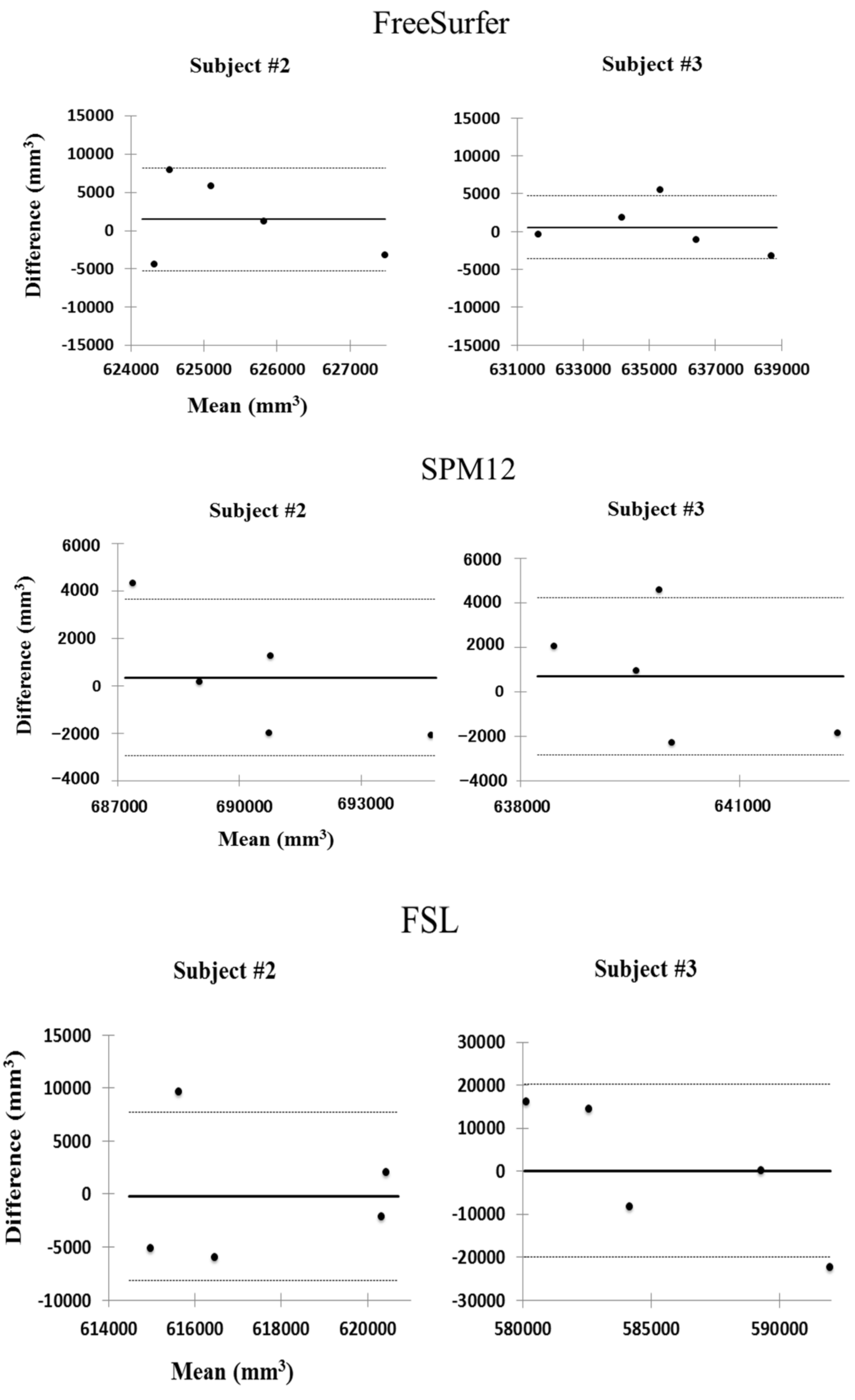
FreeSurfer
Subject #2
Subject #3
Difference (mm3)
Mean (mm3)
15000
10000
5000
0
-5000
-10000
-15000
624000
625000
626000
627000
631000
633000
635000
637000
639000
SPM12
Subject #2
Subject #3
Difference (mm3)
Mean (mm3)
6000
4000
2000
0
-2000
-4000
687000
690000
693000
638000
641000
FSL
Subject #2
Subject #3
Difference (mm3)
Mean (mm3)
15000
10000
5000
0
-5000
-10000
614000
616000
618000
620000
30000
20000
10000
0
-10000
-20000
-30000
580000
585000
590000

Supplemental Fig. S3

Bland-Altman analyses of the agreement between the motion scans with PROMO and resting scans without PROMO.

In the Bland–Altman analysis of subjects #3-7, the graphs of the total GM volumes measured by FreeSurfer, SPM12, and FSL for (A) the “side to side” motion scans with PROMO rated as adequate, (B) the “nodding” motion scans with PROMO rated as adequate, and (C) the combination of the “side to side” and “nodding” motion scans with PROMO rated as inadequate were plotted against their differences and were compared to the resting scans without PROMO.

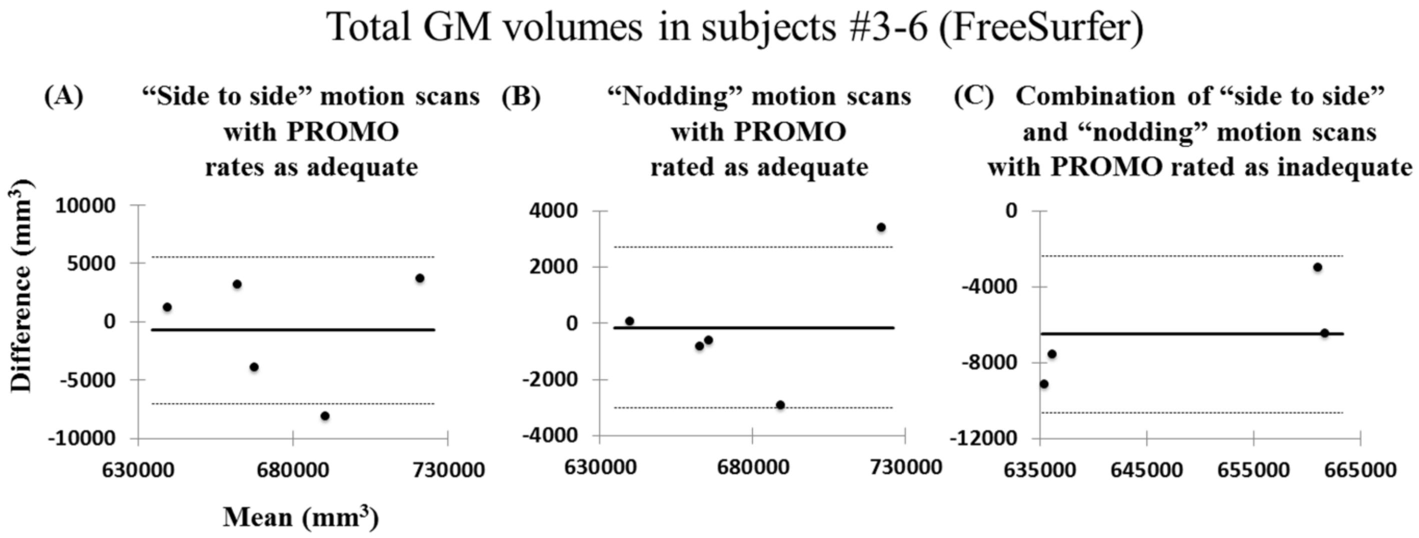

Total GM volumes in subjects #3-6 (SPM12)

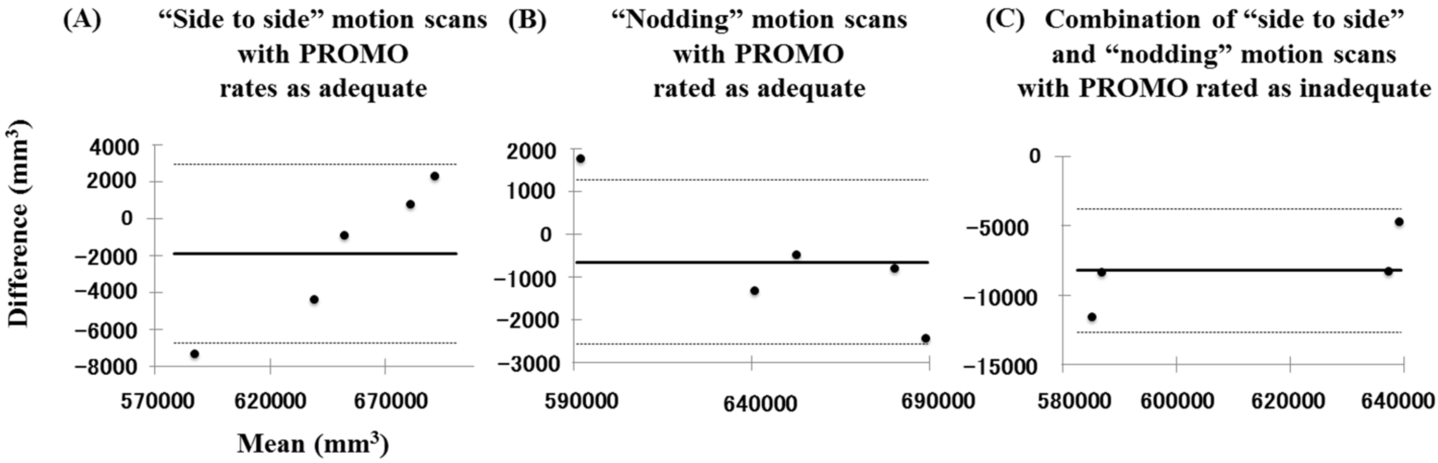

Total GM volumes in subjects #3-6 (FSL)

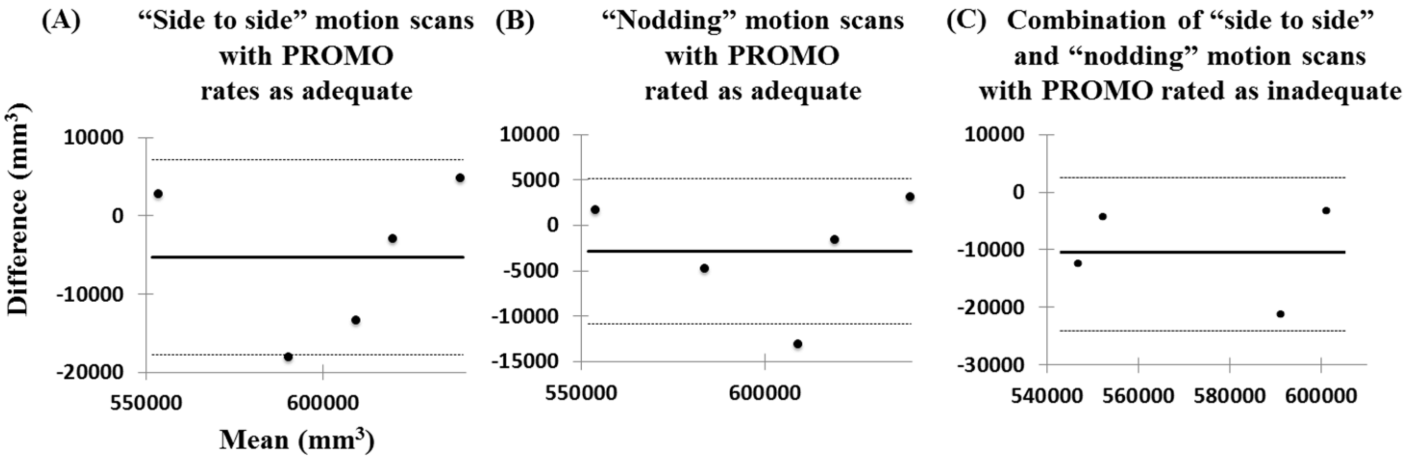

Supplemental Fig. S4

Cortical thickness difference maps between the resting scans with and without PROMO.

The cortical thickness difference maps of the left hemisphere in the subject #2 and #3 are shown. The color maps indicate the mean of thickness differences between the resting scans with and without PROMO in each vertex on the common surface space. The spatial pattern of thickness difference is incoherent across the surface atlas indicating that there are not systematic discrepancies between the resting scans with and without PROMO. The results of the right hemisphere were similar to those of the left hemisphere.

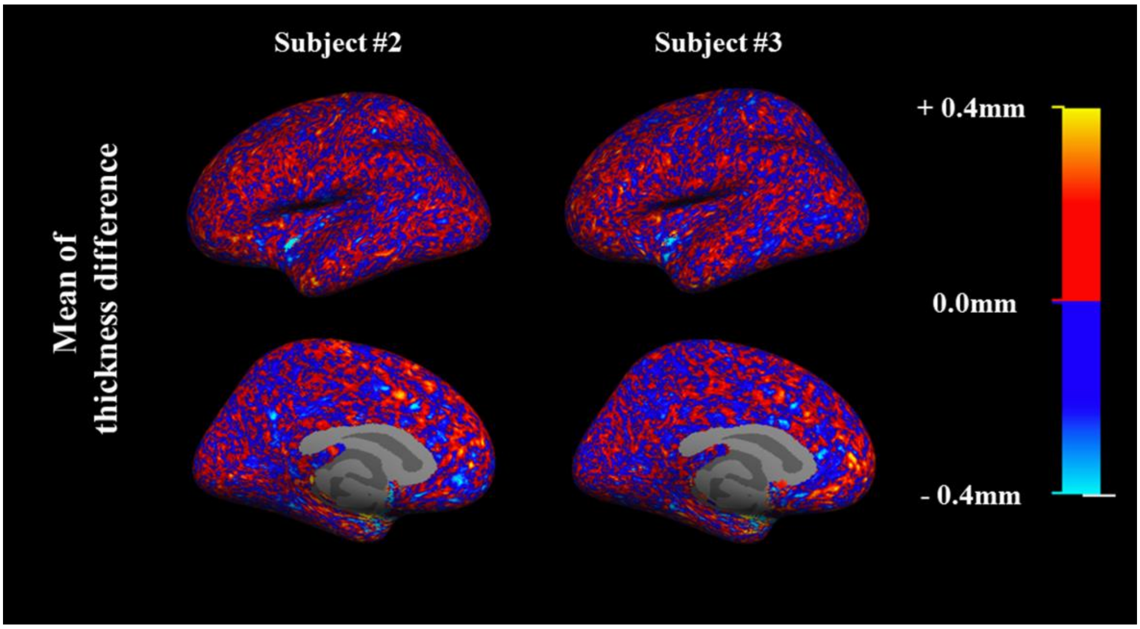

Supplemental Fig. S5

Cortical thickness difference maps between the “side to side” and “nodding” motion scans with PROMO and resting scans without PROMO.

The cortical thickness difference maps of the left hemisphere in subjects #3-7 are shown. For the “side to side” and “nodding” motion scans with PROMO rated as adequate, the spatial pattern of thickness difference is incoherent across the surface atlas, indicating no systematic discrepancies with the resting scans without PROMO. Conversely, the combination of “side to side” and “nodding” motion scans with PROMO rated as inadequate showed that the cortical thickness was underestimated, especially in the frontal cortex (arrow). The results of the right hemisphere were similar to those of the left hemisphere.

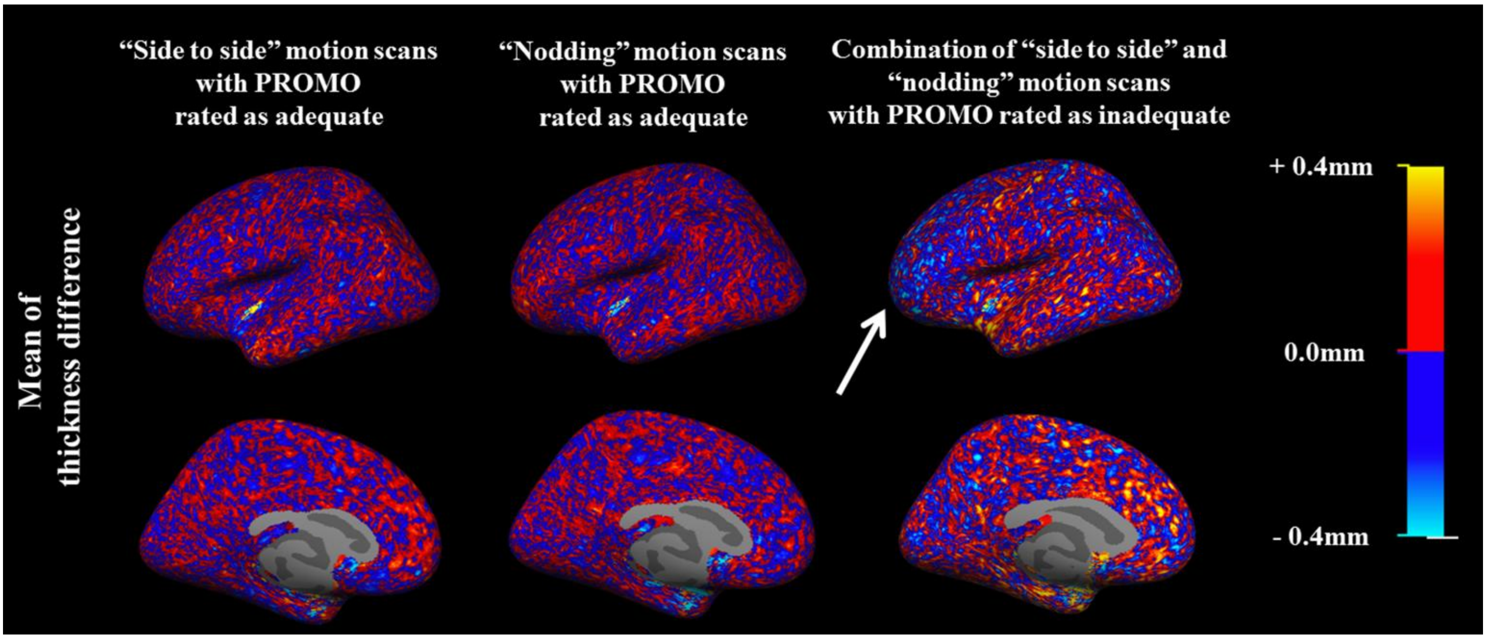

Supplement: Supplementary Figures [file srep38366-s1.pdf]
